# Gait Alterations in Two Young Siblings with Progressive Pseudorheumatoid Dysplasia

**DOI:** 10.3390/children9121982

**Published:** 2022-12-16

**Authors:** Silvia Sassi, Silvia Faccioli, Giuseppina Mariagrazia Farella, Roberto Tedeschi, Livia Garavelli, Maria Grazia Benedetti

**Affiliations:** 1 Children Rehabilitation Unit, Azienda USL-IRCCS di Reggio Emilia, 42121 Reggio Emilia, Italy; 2 PhD Program in Clinical and Experimental Medicine, Department of Biomedical, Metabolic and Neural Sciences, University of Modena and Reggio Emilia, 41100 Modena, Italy; 3 Physical Medicine and Rehabilitation Unit, IRCCS—Istituto Ortopedico Rizzoli, 40136 Bologna, Italy; 4 Department of Biomedical and Neuromotor Sciences (DIBINEM), University of Bologna, 40100 Bologna, Italy; 5 Medical Genetics Unit, Azienda USL-IRCCS di Reggio Emilia, 42121 Reggio Emilia, Italy

**Keywords:** progressive pseudorheumatoid dysplasia, gait deviations, gait analysis, muscle activity

## Abstract

Progressive pseudorheumatoid dysplasia (PPRD) is an autosomal recessive inherited skeletal dysplasia characterized by progressive non-inflammatory arthropathy affecting primarily the articular cartilage. Currently, little is known about the functional musculoskeletal aspects of these patients. In particular, an abnormal gait pattern has been described, without a clear hypothesis of the underlying causes in terms of muscular activity. This study presents the case of two siblings, 4 and 9 years old, a boy and a girl, respectively, suffering from PPRD at different stages of the disease. In addition to the clinical assessment, an instrumental gait analysis was performed. Swelling of the interphalangeal finger joints and fatigue were present in both cases. Gait abnormalities consisted of a relevant reduction in the ankle plantarflexion in the terminal phase of the gait cycle, associated with reduced gastrocnemius EMG activity and increased activity of the tibialis anterior, resulting in overloading at the initial peak of ground reaction forces. Gait anomalies observed were similar in both siblings with PPRD, although at different ages, and confirm walking patterns previously described in the literature. The calf muscle strength deficit and reduced activity during the stance phase of gait present in these two siblings indicate the typical absence of the propulsive phase. A stomping gait pattern, with the foot striking the ground hard on each step, was originally described. Further neurophysiological investigations are required to determine the origin of muscle weakness.

## 1. Background

Progressive pseudorheumatoid dysplasia (PPRD) is a genetic, non-inflammatory arthropathy with an estimated prevalence of 1/1,000,000 in the United Kingdom [1].

In most patients, the disease is manifested before the age of 6, and the onset varies between 2 and 11 years depending on different studies [1,2,3,4,5]. Generally, there are no symptoms in the neonatal period. At diagnosis, the most common signs are fatigability, gait disturbances, joint stiffness—particularly at the hip—muscle weakness and symmetrical swelling of the proximal interphalangeal joints of the hands [1,2,3,4,5]. Many patients show spinal involvement with exaggerated lumbar lordosis, thoracic kyphosis and/or scoliosis. No extra-skeletal manifestations have been reported (other than those secondary to changes in the bone). These patients have normal facial features and intelligence [1,2]. The gene responsible WISP3—wnt1-inducible signalling pathway protein 3, which maps to human chromosome 6q22—is a member of the cellular communication network (CCN) gene family [6]. CCN proteins are growth factors that regulate cell proliferation, differentiation, migration and adhesion in connective tissue [7,8,9,10].

These cases are often misdiagnosed, mimicking juvenile idiopathic arthritis or Scheuermann’s disease [3,5,11]. The diagnosis is based on clinical and X-ray findings. The radiological signs are those of a spondyloepiphyseal dysplasia (SED) with platyspondyly, a short and enlarged femoral head, enlargement of the tibial and femoral epiphyses, reduced joint spaces at the level of the hips and knees, enlargement of the epi-metaphyseal portion of the metacarpals and phalanges and osteopenia.

No destructive bone changes typical of rheumatoid arthritis are present [11], there is no inflammation of the joints, and it has been confirmed that the disorder is in connection with non-inflammatory chondropathy affecting mainly the articular cartilage [12]. Early onset of cartilage loss is a very important element in the pathogenesis of the disease. In particular, from a clinical point of view, motor weakness is present often at the onset, and the children struggle to rise from a chair and the gait become cautious and halting [11]. PPRD does not affect life expectancy; however, the progressive nature of the disease often requires replacement of the affected joint—either hip or knee—by early adulthood [1,3,11]. Muscular–skeletal involvement gradually impairs walking, although specific abnormalities are still not yet completely understood [12]. Torreggiani et al. [1] reported the findings of previous studies in which myopathy was suspected in these patients. However, electromyography (EMG) and magnetic resonance (MR) showed only minimal non-specific and non-diagnostic myopathic changes in patients with PPRD, and muscle biopsy, serum creatinine kinase and plasma lactate were normal [12]. Only one previous work has carried out an instrumental gait analysis on PPRD patients, but only from the kinematic point of view [13].

The aim of this paper was to describe the gait patterns of two siblings suffering from progressive pseudorheumatoid dysplasia at different stages of the disease by means of instrumental gait analysis including surface EMG. Recording muscle activity during gait is supposed to provide information about possible specific muscular dysfunction.

## 2. Case Presentation

Two siblings, aged 4 and 9, a female and a male, were evaluated in this study. Disease onset was seen within the first 4 years of life. The diagnosis of PPRD was not immediate in the first-born, as juvenile idiopathic arthritis was suspected, but it was later confirmed with molecular analysis. The sequencing analysis revealed in both the presence of the variant c.156C>A, in homozygosity, in the *WISP3* gene, which, at the protein level, determines the p.Cys52Ter variant. The variant c.156C>A, segregated from the parents, both heterozygous carriers, is classified in the reference databases as a pathogenetic variant (ClinVar allele ID: 21420) and is known in the scientific literature [3].

**Figure 1:**
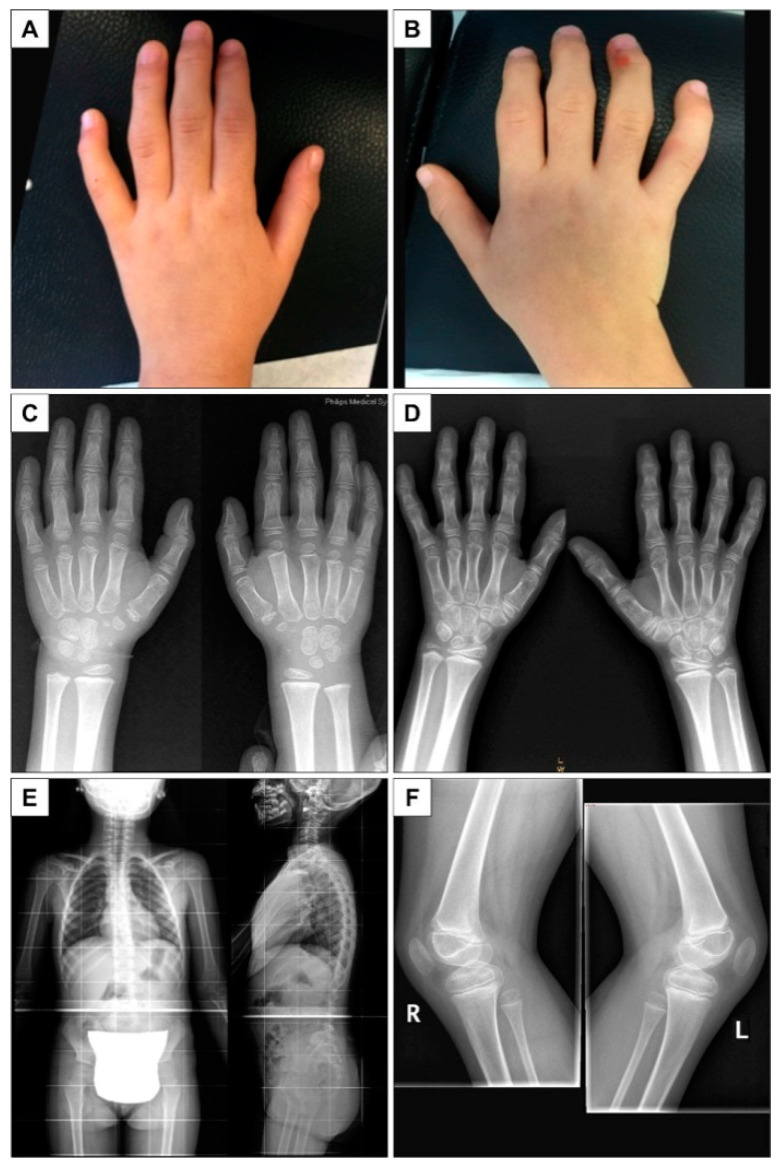
Clinical and X-ray images of the two siblings. Left: boy, right: girl. (**A**,**B**) First patient, age 6 years: swelling of the interphalangeal finger joints. (**C**) Second patient, age 4 years. Hand X-rays: enlargement and flattening of distal extremities of the proximal phalanges and enlargement and flattening of the proximal extremities of the middle phalanges, flat phalangeal epiphysis. Osteopenia. Bone age: carpus: 5 years, phalanges: 4 years 6 months (Greulick and Pyle method). (**D**) First patient, age 6 years. Hand X-rays: enlargement of proximal and distal phalangeal extremities, flat phalangeal epiphysis, crowded carpal bones. Osteopenia. Bone age: carpus: between 7 years 6 months and 8 years 6 months, phalanges: 5 years 6 months (Greulick and Pyle method). (**E**) First patient, age 6 years. Spine X-rays: slight platyspondily. Dorso-lumbar kyphoscoliosis. Bilateral coxa valga. (**F**) First patient, age 6 years 2 months. Knee X-rays: no double patella. Osteopenia.

For both children, abnormal gait was the first symptom. It was characterized by waddling with a broadened stride, as previously described [2]. Another early symptom was swelling of the interphalangeal finger joints (Figure 1). Muscle weakness was one of the main symptoms. At the onset, joint pain was absent; however, progressive functional impairment was present in both during walking. The movement of the hip joint was more restricted in the girl, particularly in rotation, and knee ROM was restricted by 5 degrees in extension as compared to the boy (Table 1). Passive dorsal and plantar ankle motion was normal, but both children had severe plantar flexion strength deficits. All limitations were symmetrical. At the time of the analysis, they were not yet using walking aids.

Gait analysis with an eight-camera stereophotogrammetric system (Vicon 612, Oxford Metrics, Oxford, UK) and two forceplates (Kistler, Winterthur, Switzerland) was performed to evaluate lower limb joint kinematics and kinetics. Passive reflective markers were attached with double-sided adhesive tape to anatomical reference points, according to an established marker set and gait protocol, the Total3DGait Protocol [14]. The markers were positioned at the sacrum, anterior superior iliac spines, greater trochanters, lateral femoral condyles, heads of fibula, lateral malleoli, heads of the first and fifth metatarsals and heels. After the subjects of the study were assessed in the standing position, they were asked to walk on a pathway of approximately 9 meters in length. The subjects walked barefoot, at a comfortable speed. Five to seven consecutive trips were recorded. The spatiotemporal data collected from gait analysis were the stance time (%), swing time (%), double stance time (%), cycle time (s), stride length (cm), cadence (steps/min) and speed of progression (cm/s). The mean value of all the gait trials was considered.

Kinematics (joint rotations) and kinetics (moments and power) in the three planes of space were registered for the hip, knee and ankle–foot complex. All the trials are reported in the figures for qualitative analysis. 

Muscle activities were simultaneously recorded using surface electromyography (EMG) (Wave Wireless, COMETA, Milan, Italy) with a sampling frequency of 500 Hz. Surface EMG of the rectus femoris, hamstrings, tibialis anterior and medial gastrocnemius at both limbs was also performed. Data were qualitatively compared with a sample of patients matched for age and sex [13]. Clinical assessment and gait analysis were performed as clinical routine, with the informed consent of the parents of the patients. 

Analysis of spatiotemporal parameters in both siblings showed a reduction in speed, essentially due to reduced stride length in both children (Table 2) and an arrhythmic gait for the girl.

**Figure 2:**
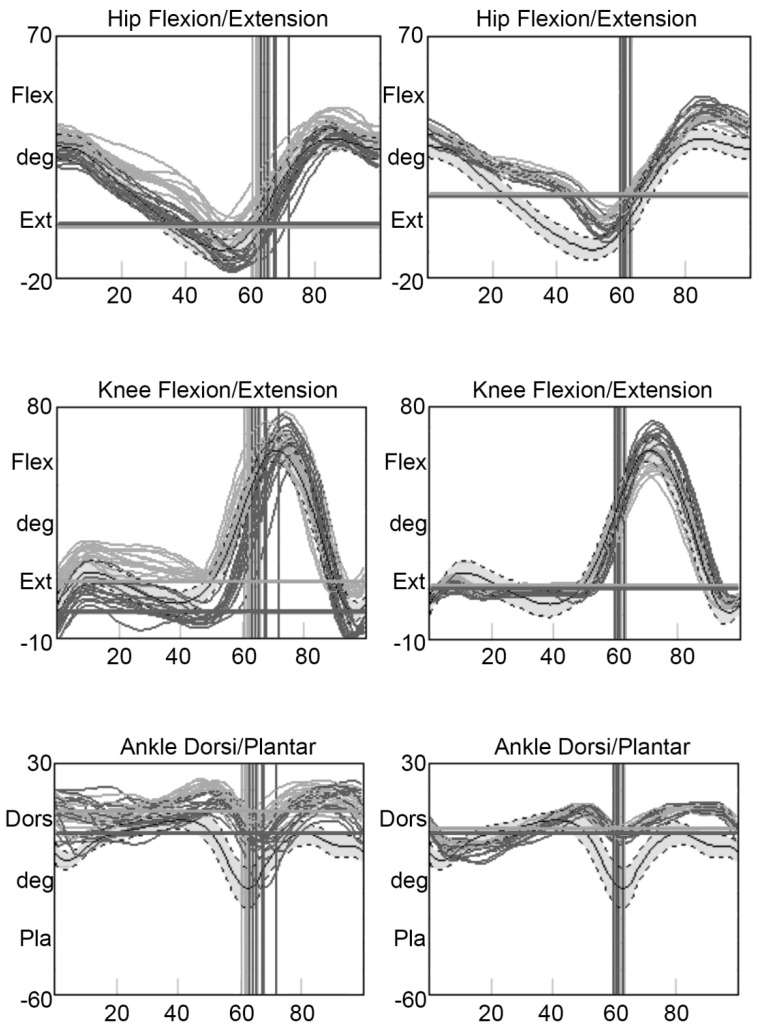
Hip, knee and ankle kinematics in the sagittal plane of the two patients. Grey lines correspond to the right side, black lines to the left side. The vertical lines indicate the toe-off, the horizontal line the standing joint position. The grey band represents the control group (mean ± 1 SD). Left: boy, right: girl.

From a kinematic point of view, in both children, the extension of the hip at the end of the stride was reduced, with a typical incisura in the midstance, symmetrical in the girl and present only at the right side for the boy (Figure 2).

The knee extension pattern at the loading response phase was reduced in the girl, while the brother showed a more regular pattern, but clearly asymmetric between the two sides. Plantar flexion at terminal stance was completely missing in both patients.

**Figure 3:**
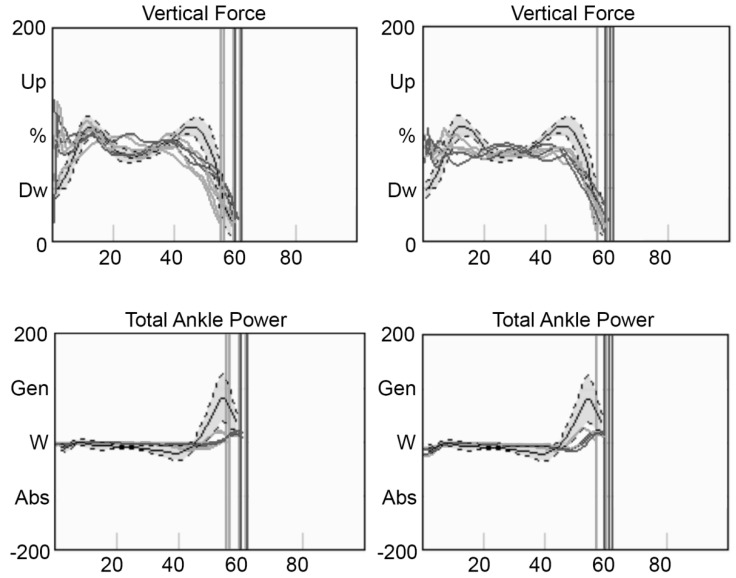
(**Top**): vertical component of ground reaction forces of the two patients. (**Bottom)**: ankle power in the sagittal plane. Grey lines correspond to the right side, black lines to the left side. The vertical lines indicate the toe-off, the horizontal line the standing joint position. The grey band represents the control group (mean ± 1 SD). (**Left**): boy, (**right**): girl.

The vertical component of the reaction forces showed a very high impact-to-ground peak after initial contact. Ankle power was markedly reduced during the push-off phase (Figure 3).

**Figure 4:**
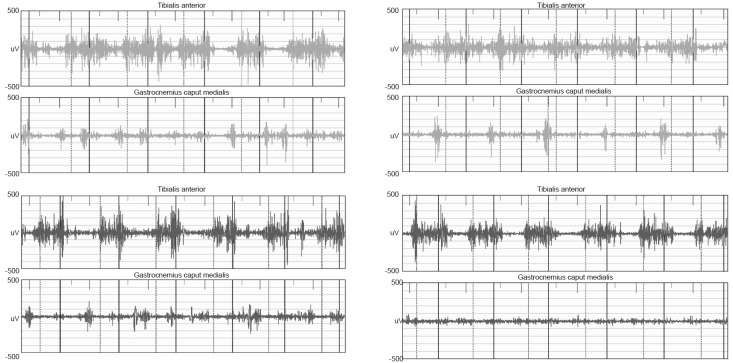
Surface EMG of right limb (**up**) and left limb (**down**) of the two siblings. The raw signal of tibialis anterior and medial gastrocnemius is reported along successive gait strides. The solid vertical line indicates the heel strike and the dashed line indicates the toe-off of consecutive strides. (**Left**): boy, (**right**): girl.

This finding is consistent with the activity of the gastrocnemius, which was reduced in both patients during the terminal stance and completely absent at the left side for the girl (Figure 4). Conversely, the anterior tibial muscle tended to show protracted action throughout the support phase at the right side in both patients. Furthermore, the boy presented out-of-phase activity of the gastrocnemius at the end of the swing phase bilaterally. 

## 3. Discussion and Conclusions

The gait analysis of the two siblings with PPRD in the present study confirmed the presence of gait abnormalities. Examination of the spatiotemporal parameters revealed a comparable gait in both children: according to a previous study [13], the gait is slow, and, in the older patient, it is also arrhythmic. Abnormalities in the kinematics of the hip, knee and ankle in the sagittal plane are similar to those already described too [13], with a reduced extension of the hip, abnormal knee flexion/extension pattern during stance phase and, in particular, reduced plantar flexion in the terminal stance at the ankle.

Pain, joint stiffness, fatigue and muscular weakness are all factors called into question to explain these anomalies [13]. Considering that, in these two patients, clinical assessment did not evidence particular joint range of motion restriction in the sagittal plane, and no pain was complained of by the patients, fatigue and muscle weakness can be considered responsible for the gait deviations. Generalized muscle weakness has been described as a specific sign of the disease [2,12] and can be amplified by early fatigue, which is described as one of the first symptoms of PPRD [3], resulting in a slow gait. However, focal weakness of the gastrocnemius, a key muscle during gait, can be responsible for the lack of the propulsive phase, as evidenced by the ankle kinematics and the kinetics—and as an original finding in the present study—and by the reduced gastrocnemius EMG activity during the terminal stance. Reduced hip extension can also be linked to the lack of the propulsive phase of the gait [15]. The marked activation of the anterior tibial muscle during stance at the right side of both siblings appears compensatory, probably used to aid body progression during gait, or to guarantee balance in the frontal plane through the supination when the contralateral limb is in the swing phase. More difficult to explain is the peak force upon heel strike presented by both siblings. This stomping gait, with the foot striking the ground hard on each step, highlights difficulty in controlling muscles when preparing the foot to come into contact with the ground, and it is similar to a sensory ataxic gait. It features an abrupt impact against the ground, which could cause overloading at the upper joints. Since it has been reported [3] that most patients with PPRD will require joint replacement in early adulthood—particularly of the hip and knee joints—treatment in childhood and adolescence should focus on sparing these joints and correcting the pattern of joint overload upon impact with the ground.

The pattern described in these two patients seems not very different from the kinematic gait pattern in children and adolescents with juvenile idiopathic arthritis, in which a slow gait, an increased pelvic tilt and hip flexion during the whole gait cycle, limited hip extension and limited plantar flexion in the ankle were reported [16]. As stated, these similarities can be interpreted as compensatory changes in response to arthralgia and limb deformations. Furthermore, the weakness and atrophy of the calf muscles could be attributed even to the arthrogenic muscle inhibition (AMI), a presynaptic reflex inhibition of the periarticular musculature elicited by abnormal input to the joint, such as swelling and pain [17], although no pain or ankle impairment that could account for this condition was present in the two children.

In conclusion, with caution due to the study being focused on two patients only, we can state that the alterations in gait observed in the case presented here are similar in both siblings with PPRD, although at different ages, and confirm walking patterns previously described [11]. As original findings of the present study, reduced action of the ankle plantaflexors and increased activity of the tibialis anterior were reported during gait. This finding can be of clinical relevance from a rehabilitation point of view. The strengthening of the calf muscles, associated with appropriate shoes, shock-absorbing insoles or dynamic ankle–foot orthoses (such as carbon fiber leaf spring ankle foot orthoses), could help in controlling the abnormal gait pattern. Furthermore, recent techniques of rehabilitation using motion observation and imagery acting on neural systems, such as the corticospinal tracts and spinal reflex circuits relieving AMI, could be effective in restoring motor function in these patients.

The present study has some limitations, since no electrophysiological study was conducted in order to evaluate neuromuscular function in these two patients. Moreover, accurate muscular testing by means of a dynamometer was not performed. Future research in larger samples of affected individuals at various ages, consistent with the fact that this is a rare disease, is desirable to better understand the origin of muscle weakness. This should take into account a possible muscle electrodiagnostic examination and an accurate muscle strength assessment as an extension of the neurological evaluation.

## Figures and Tables

**Table 1:** Anthropometric and clinical data of 2 siblings affected by PPRD.

	Boy	Girl
Age	4	9
Height (cm)	101	114
Weight (kg)	16	20
BMI (kg/m^2^)	15.68	15.39
PROM—Left/Right Hip (deg)	Ext Rot 60; Int Rot 45 (bil)	Ext Rot 30/40; Int R 30/30
PROM—Left/Right Knee (deg)	Complete	−5 (bil)
PROM—Left/Right Ankle (deg)	20–50	20–50
Popliteal Angle—Left/Right (deg)	30 (bil)	20 (bil)
Pain	No	No
Inter-Phalangeal Deformity	Yes	Yes
Fatigability	Yes	Yes

**Table 2:** Spatiotemporal parameters of the two siblings affected by PPRD.

	Boy		Girl	
	Left Side	Right Side	Left Side	Right Side
1 Double Support (%)	12.6 ± 1.5	13.1 ± 3.1	11.6 ± 1	11.7 ± 1.3
Single Support (%)	39.7 ± 2.6	35.3 ± 3.9	38.6 ± 1.3	38.4 ± 1.5
2 Double Support (%)	13.2 ± 2.3	12.9 ± 1.3	11.7 ± 0.8	11.8 ± 1
Cadence (steps/min)	75.6 ± 4.1	75.1 ± 3.9	71.2 ± 4.6	72.0 ± 5.3
Cycle Time (s)	0.80 ± 0.05	0.80 ± 0.04	0.85 ± 0.05	0.84 ± 0.06
Speed (cm/s)	90.4 ± 9.3	91.0 ± 10.8	96.1 ± 11.0	96.6 ± 10.2
Stance Time (%)	65.2 ± 2.6	61.2 ± 1.9	61.9 ± 0.8	61.1 ± 1.5
Stride Length (cm)	71.6 ± 3.9	72.6 ± 5.7	80.8 ± 4.5	80.4 ± 3.7

## Data Availability

The dataset analyzed during the current study is available from the corresponding author on reasonable request.
